# Body weight and 25-hidroxyvitamin D follicular levels: a prospective study of women submitted to in vitro fertilization

**DOI:** 10.5935/1518-0557.20160029

**Published:** 2016

**Authors:** Vitor AS Deriquehem, Roberto A Antunes, Mila W Reginatto, Ana C Mancebo, Patricia Areas, Enrrico Bloise, Maria do Carmo B de Souza, Tania M Ortiga-Carvalho

**Affiliations:** 1 Laboratory of Translational Endocrinology, Carlos Chagas Filho Biophysics Institute, Federal University of Rio de Janeiro.; 2 Teaching Maternity Hospital, Federal University of Rio de Janeiro. Rio de Janeiro/RJ, Brazil.; 3 Fertipraxis Clinic, Rio de Janeiro/RJ, Brazil.

**Keywords:** Vitamin D, Calcitriol, Follicular fluid, BMI, IVF/ICSI

## Abstract

**Objective:**

Vitamin D deficiency has been largely related to infertility in animals. However, data demonstrating a direct association between hypovitaminosis D and infertility in humans are still conflicting. Increased body weight and an elevated body mass index (BMI) are known for their association with infertility. Therefore, this study attempted to verify whether increases in body weight and the BMI were associated with lower 25-hidroxyvitamin D [25(OH)D3] levels in the follicular fluid (FF) of patients treated for infertility with intracytoplasmic sperm injections (ICSI). This study aimed to assess the FF levels of 25(OH)D3 in women submitted to ICSI and correlate these levels with the different body weight and BMI values observed in the enrolled cohort.

**Methods:**

The FF aspirates of 199 patients submitted to ICSI were collected after oocyte aspiration to check whether FF 25(OH)D3 levels were associated with weight regardless of the etiology of infertility. Chemiluminescent assays were used to assess FF 25(OH)D3 levels. The etiology of infertility was defined based on patient clinical history and follow-up.

**Results:**

The patients enrolled in the study were divided into three groups according to their FF 25(OH)D3 levels, as follows: a) deficient (n=71; <20 ng/ml); b) insufficient (n=64; 21< 25(OH) D3>29 ng/ml); and c) sufficient (n=56 >30ng/ml) levels. Patients with lower FF 25(OH)D3 levels had a greater mean weight (64.1kg) when compared to patients with higher 25(OH)D3 levels (60.7kg), p<0.01. No differences were observed in terms of age or etiology of infertility.

**Conclusion:**

The body weight of the individuals with FF 25(OH)D3 deficiency measured in single follicles was significantly higher regardless of the etiology of infertility. Further epidemiologic and molecular studies are required to verify whether the amount of follicular 25(OH)D3 affects the outcome of IVF procedures.

## INTRODUCTION

Vitamin D plays a major role in bone and calcium metabolism, in addition to acting in the regulation of the cardiovascular and immune systems. Humans obtain their daily levels of vitamin D mainly by endogenous biosynthesis induced by exposure to ultraviolet B (UVB) radiation, in concert with a small contribution from the intake of different food sources. Cholecalciferol (vitamin D3) and ergocalciferol (vitamin D2) are hydroxylated in the liver, resulting in 25-hidroxyvitamin D or 25(OH)D3. In the kidneys, 25(OH)D is metabolized as the active form -calcitriol (1,25-dihidroxyvitamin D) - or as an inactive metabolite - 24,25-hidroxyvitamin D. Vitamin D deficiency is defined as serum 25(OH)D3 levels below 20 ng/ml, whereas sufficient levels are defined by values greater than 30 ng/ml (LeBlanc *et al.*, 2015).

The role of vitamin D in fertility and ovarian function is still largely unexplored. Vitamin D receptor (VDR) expression has been observed in the ovaries of animal models, more specifically in the granulosa cells and follicles (Brannian *et al*., 2009). There is a direct association between vitamin D levels and fertilization rates in different animal models (Panda *et al*., 2001; Luk *et al*., 2012). In humans, the role of vitamin D in fertilization, embryo quality and implantation is still controversial (Terushkin *et al*., 2010; Aleyasin *et al*., 2011; Rudick *et al*., 2012; Aflatoonian *et al*., 2014; Paffoni *et al*., 2014; Polyzos *et al*., 2014). Several authors postulated that implantation and clinical pregnancy rates were lower in patients with serum 25(OH)D3 < 20ng/ml (Luk et al., 2012; Paffoni et al., 2014; Perez-Lopez *et al*., 2015). Better in vitro fertilization (IVF) outcomes were related to increased follicular fluid (FF) levels of 25(OH)D3 (Ozkan *et al*., 2010; Farzadi *et al*., 2015). One meta-analysis described an association between lower live birth rates and lower serum 25(OH)D3 levels in women submitted to IVF, with no differences in clinical pregnancy rates (Lv *et al*., 2016), suggesting that serum 25(OH)D3 was key to pregnancy progression. However, several studies failed to find correlations when clinical pregnancy rates were compared against 25OH)D3 serum levels in patients submitted to IVF (Aleyasin et al., 2011; Polyzos et al., 2014; Fabris *et al*., 2014), highliting the need for more studies investigating the impact of 25(OH)D3 in reproductive outcomes.

In terms of obstetric outcomes, 25(OH)D3 deficiency has been associated with increased risk of preeclampsia, gestational diabetes, and low birth weight (Aghajafari *et al*., 2013; Tabesh *et al*., 2013; Theodoratou *et al*., 2014).

Body weight and the body mass index (BMI) are important factors associated with hypovitaminosis D and infertility (Wortsman *et al.*, 2000; Reis *et al*., 2009). A study described a direct association between 25(OH)D3 levels and the BMI, weight, and the waist-hip ratio of a group of women living in the same latitude followed for 30 years. Another study demonstrated that obese and overweight patients had lower 25(OH)D3 levels versus patients with normal weight (Tosunbayraktar *et al*., 2015). Interestingly, the obese patients had lower levels of 25(OH)D3 than the overweight individuals. Vitamin D deficiency is highly prevalent among infertile women and has been inversely correlated with the BMI and positively correlated with exposure to sunlight, but data comparing cause of infertility and height were conflicting. The maternal BMI is also greater in pregnant patients with lower 25(OH)D3 levels.

As for the newborn, umbilical cord blood 25(OH)D3 levels varied according to the season of birth, latitude, and length of the newborn, but not with weight at birth, adiposity, head circumference, or maternal 25(OH)D3 levels. However, maternal 25(OH)D3 levels were positively associated with infant weight at birth related to gestational age. Even though Tian found a positive association between maternal vitamin D levels and infant weight at birth, vitamin D supplementation for pregnant women with low serum levels of 25(OH)D3 failed to improve birth weight outcomes. In fact, the benefits associated with gestational vitamin D supplementation are debatable, and more research is warranted (Dalgard *et al*., 2016; Dressler *et al*., 2016).

In a nutshell, it is still fairly unclear whether FF levels of 25(OH)D3 are associated with the BMI or the weight of infertile women, and if infertility can be correlated at all with 25(OH)D3 levels in the FF. Therefore, this study aimed to assess whether there are similarities in the serum and follicular levels of 25(OH)D and to analyze the FF levels of 25(OH)D3 in women with different BMIs submitted to ICSI.

## MATERIALS AND METHODS

### Patients

The patients enrolled in this study underwent ICSI at the Fertipraxis Center for Human Reproduction, a clinic certified by the Brazilian health surveillance authority (ANVISA) and the Latin American Network of Assisted Reproduction (REDLARA). The local Ethics Committee approved the study protocol; all enrolled patients gave written consent before joining the study.

### Follicular fluid aspiration

All female patients referred to oocyte aspiration between July of 2012 and November of 2014 had a sample of follicular fluid collected on the same day of oocyte pickup. The samples were collected using a sterile vacuum system set to 90 mmHg with a 17G Wallace® aspiration needle. In order to obtain a pure, single follicular fluid sample, without the contamination of blood or fluid from other follicles during aspiration, the aspiration procedure was initiated in the first follicle measuring more than 17mm. As soon as the follicle was emptied, the aspiration needle was removed from the ovary and all the fluid content that was still in the circuit of the vacuum system was transferred to a sterile storage container. Then, the container with the sample was disconnected from the vacuum system and taken to the lab. At the lab, an embryologist searched the fluid to retrieve its oocyte and, after the oocyte was removed, the follicular fluid was placed in a sterile cryo tube and immediately frozen in liquid nitrogen. A new storage container was then connected to the vacuum system and the oocyte aspiration resumed, this time pooling all the remaining follicular fluid aspirated in order to retrieve as many oocytes as possible. For purposes of analysis, all samples were thawed and centrifuged at 1500 g for 15 minutes. Patients submitted to ovarian hyper stimulation protocols with aromatase inhibitors were excluded. The included individuals were on protocols with either recombinant FSH (fresh) or r FSH plus purified HMG (Urinary Menopausal Gonadotropins, us + lush) associated to GnRH antagonists.

### Vitamin D level measurements

FF 25(OH)D3 levels were assessed with a chemiluminescent immunoassay (Elecsys total vitamin D -Roche Diagnostics, Brazil) at the Laboratory of Molecular and Translational Endocrinology, Department of Medicine, Paulista School of Medicine, Federal University of São Paulo (Unifesp/EPM), São Paulo, Brazil. Inter-assay and intra-assay variation was 8.3% and 9.1%, respectively.

### BMI calculation

The BMI was calculated based on the height and weight measured for each patient. The causes of infertility were described in the patients' charts.

### Statistics

The data were presented in the form of mean ± standard deviation. The D'Agostino-Pearson test was used to test for normality. One-way analysis of variance (ANOVA) and the Bonferroni multiple comparisons test were used in the analysis of body weight, BMI, age, and 25(OH)D3 levels one. The Bland-Altman plot and Pearson's correlation coefficient were used to validate the association between serum and FF 25(OH)D3 levels. Statistical analyses were performed using the Graphpad Prism 6 Software (GraphPad Software, Inc., San Diego, CA, USA). Significance was attributed to differences with a *P*<0.05.

## RESULTS

**Figure 1:**
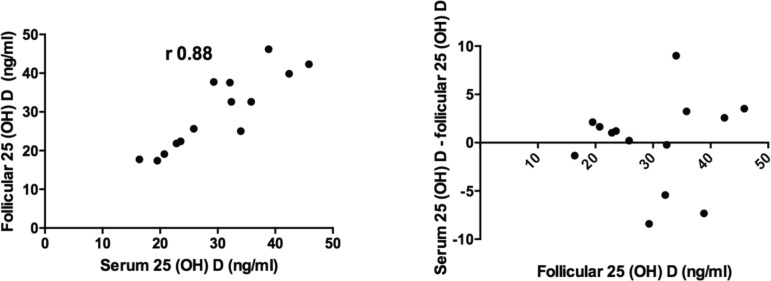
Follicular and Serum 25(OH) levels of 14 patients. Pearson’s correlation coefficient and Bland-Altman plots.

One hundred and ninety-nine patients were enrolled in this study. Figure 1 shows strong positive correlations between the serum and FF 25(OH)D3 levels of 14 randomly selected patients. The Bland-Altman plot showed a bias close to zero (0.12), confirming the strength of the correlation.

**Figure 2:**
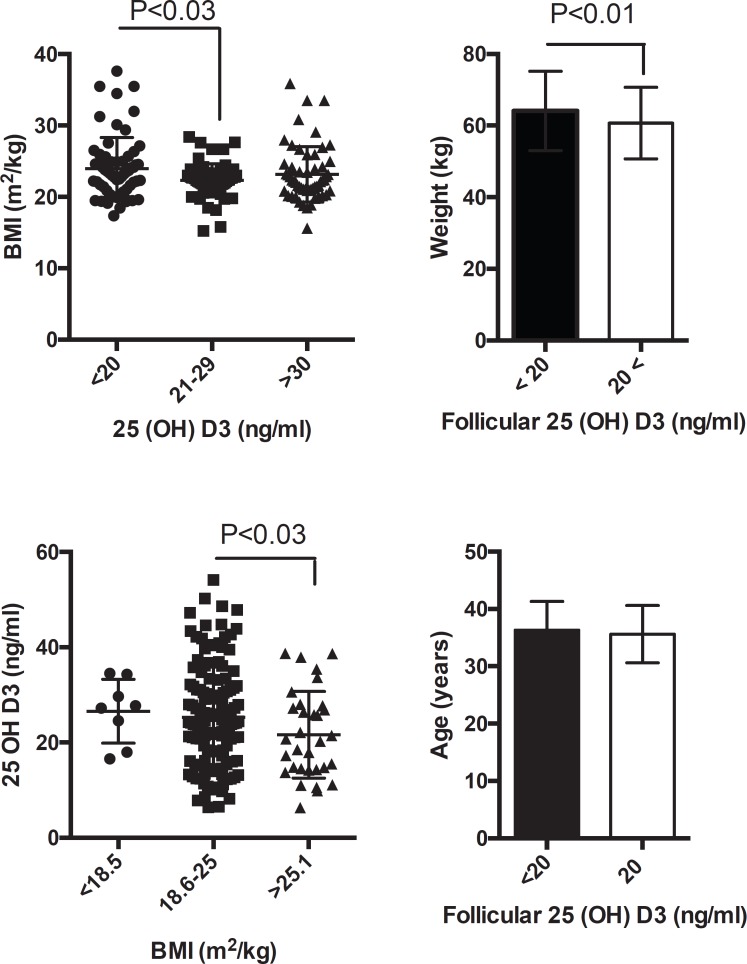
Body mass index (m2/kg) and follicular levels of 25 (OH) D3 (ng/ml). Individual patients are shown. Lines represent mean values and standard deviation

Figure 2 shows the patient BMIs sorted into groups based on 25(OH)D3 FF levels. Four percent of the patients had low BMIs (<18.5kg/m2), 67% had normal BMIs (20 to 25 kg/m2), and 19% were overweight or obese (>25kg/m2). Patients with lower levels of 25(OH)D3 (<20 nl/ml) had higher BMIs (*P*<0.03). When the patients were sorted based on their BMIs, the group with the higher BMIs (>25kg/m2) had lower levels of FF 25(OH)D3. The patients were then divided into two groups according to whether they had FF levels of 25(OH)D3 lower or higher than 20 ng/ml.

**Figure 3:**
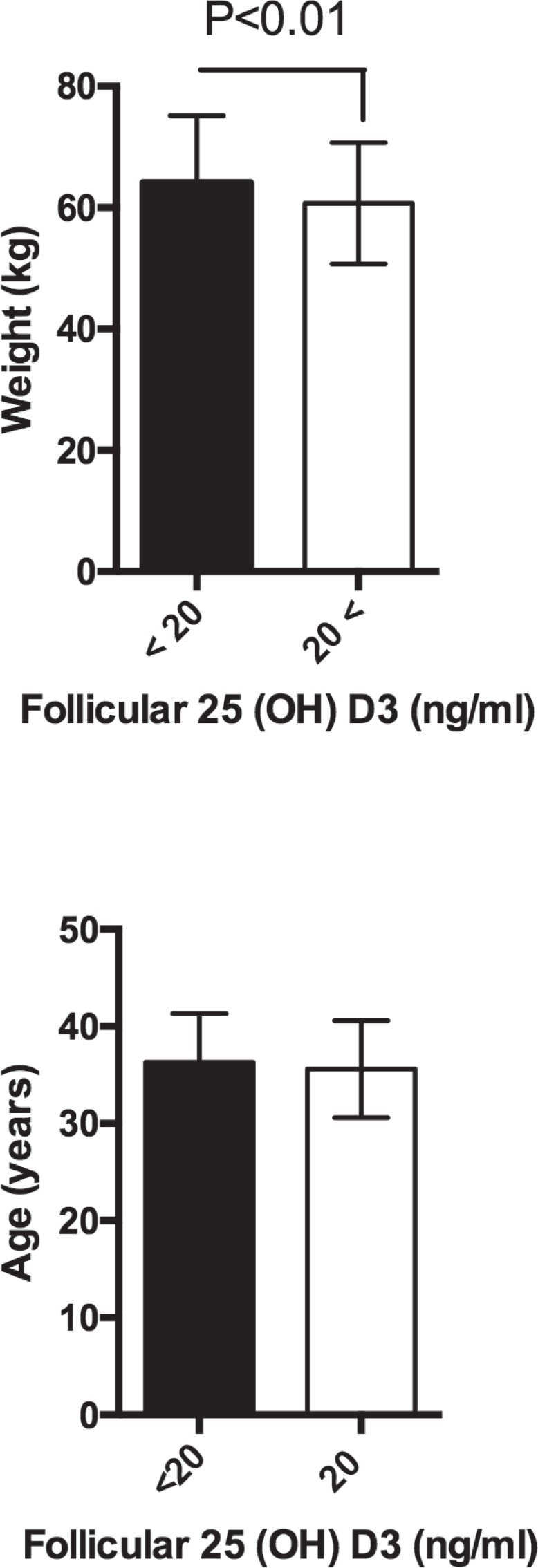
Weight (kg) and age (years) of patients with >20ng/ml and <20ng/ml of 25(OH) D3. Bars represent mean values and standard deviation.

The patients enrolled in the study had a mean age of 35.9±4.2 years. The youngest was 22 and the oldest 48 years old. Seventy-one women had 25(OH)D3 follicular fluid levels below 20ng/ml, with a mean value of 13.2±4.1 ng/ml, and 128 women had follicular fluid levels of 25(OH)D3 above 20 ng/ml, with a mean value of 30.7±8.3 ng/ml. No differences were observed in the age and total doses of recombinant FSH given to both groups. The FF 25(OH)D3 levels seen in both groups were significantly different (*P*<0.05). The causes of infertility were distributed almost equally (*P*>0.05), with unexplained reasons and reduced ovarian reserve ranking as the most prevalent (35% vs. 33.5%), followed by male factor infertility (30% vs. 24.2%), tubal factor infertility or endometriosis (20% vs. 15.6%), mixed factors (7.5% vs. 7.8%), and others (7.5% vs. 18.9%). Patients with FF 25(OH)D3 deficiency were heavier than their counterparts with higher levels of vitamin D (64.1±12.1 kg vs. 60.7± 10.5 kg, *P* <0.01, Table 1 and Figure 3).

**Table 1:** Weight, age, recombinant FSH doses and causes of infertility

	25 OH D3 (ng/ml) <20	25 OH D3 (ng/ml) >20	*P*
n	71	128	
Weight (Kg)	64.1kg±12.1	60.7kg±10.5	<0.05
Age (years)	36.3	35.6	>0.05
Causes of Infertility (%)			
Unexplained/reduced ovarian reserve	35	33.5	>0.05
Male factor infertility	30	24.2	>0.05
Tubal factor infertility/endometriosis	20	15.6	>0.05
Mixed	7.5	7.8	>0.05
Others	7.5	18.9	>0.05

## DISCUSSION

This study looked into a cohort of Brazilian infertile women to see whether their FF 25(OH)D3 levels would vary depending on their weights and BMIs. The complex ethnic makeup of the group prevented the description of differences based on ethnicity. Therefore, the patients were divided into groups having 25(OH)D3 follicular levels as basis. First we checked whether follicular vitamin D levels reproduced serum vitamin D levels. Few studies described an equivalence between follicular fluid (FF) and serum levels of 25(OH)D3 (Ozkan *et al*., 2010; Aleyasin *et al*., 2011; Rudick *et al*., 2012; Firouzabadi *et al*., 2014). Data analysis by Pearson's correlation coefficient and Bland-Altman plots confirmed the existence of such equivalence, and follicular levels were then used to stratify patients in this study.

The American Society of Endocrinology categorizes subjects based on their 25(OH)D3 levels: 20, 21-29, and 30 ng/ml are the cutoffs for deficiency, insufficiency and sufficiency, respectively (Holick *et al*., 2011). The correlation between 25(OH)D3 deficiency and obesity has been reported (Wortsman *et al.*, 2000; Harel *et al*., 2011). In our cohort, most of the subjects had normal body weights and BMIs of less than 25m2/kg; decreases in follicular 25(OH)D3 levels were observed among overweight and obese individuals (>25.1 m2/kg). Obesity is known to affect male and female fertility, and many authors have explored this subject (Brannian *et al*., 2009; Dressler *et al*., 2016). However, it is difficult to separate the effects of obesity alone from factors associated to sedentarism and hormone levels altered by weight gain.

Controversy still looms over the categorization of 25(OH)D3 levels as sufficient or insufficient. The capture of 25(OH)D3 by adipocytes or the idea that adipocytes act as a reservoir of calcitriol may be true; yet, the two theories still lack supporting evidence. Patients presenting with 25(OH)D3 serum levels of less than 20 ng/ml are currently considered deficient. The groups in this study were divided based on this threshold, and each was evaluated for their differences. The age and prescribed dose of recombinant FSH were not different, but patients with 25(OH)D3 deficiency were heavier than the individuals with levels greater than 20 ng/ml. The association between weight and FF levels of 25(OH)D3 may be affected by multiple factors, such as a sedentary lifestyle and/or a poor diet. Another plausible explanation is the molecular abduction of vitamin D by adipose tissue discussed above. One of the strengths of our study is the fact that each sample corresponds to one pre-ovulatory follicle from each woman. However, the study's limitations include the small number subjects enrolled and the lack of a lifestyle assessment questionnaire.

The two groups were also analyzed for infertility. Unexplained infertility and reduced ovarian reserve were the main reported causes of infertility, followed by male factor infertility and tubal factor infertility/endometriosis. Patients with more than one factor were classified as "Mixed," since a single factor could not be directly associated with the observed follicular levels of 25(OH)D3. Both groups had equal distributions of causes of infertility, suggesting the absence of an association with the metabolism of vitamin D.

To sum up with, women with deficient levels of 25(OH)D3 measured in single follicles were significantly heavier regardless of their infertility statuses. The level of follicular 25(OH)D3 within individual follicles may possibly impact the outcome of IVF procedures. However, further epidemiologic and molecular studies are warranted to better understand the impact of FF 25(OH)D3 levels in reproduction.

## References

[r1] Aflatoonian A, Arabjahvani F, Eftekhar M, Sayadi M (2014). Effect of vitamin D insufficiency treatment on fertility outcomes
in frozen-thawed embryo transfer cycles: A randomized clinical
trial. Iran J Reprod Med.

[r2] Aghajafari F, Nagulesapillai T, Ronksley PE, Tough SC, O'Beirne M, Rabi DM (2013). Association between maternal serum 25-hydroxyvitamin D level and
pregnancy and neonatal outcomes: systematic review and meta-analysis of
observational studies. BMJ.

[r3] Aleyasin A, Hosseini MA, Mahdavi A, Safdarian L, Fallahi P, Mohajeri MR, Abbasi M, Esfahani F (2011). Predictive value of the level of vitamin D in follicular fluid on
the outcome of assisted reproductive technology. Eur J Obstet Gynecol Reprod Biol.

[r4] Brannian J, Eyster K, Greenway M, Henriksen C, Teslaa K, Diggins M (2009). Progressive obesity leads to altered ovarian gene expression in
the Lethal Yellow mouse: a microarray study. J Ovarian Res.

[r5] Dalgard C, Petersen MS, Steuerwald U, Weihe P, Grandjean P (2016). Umbilical Cord Serum 25-Hydroxyvitamin D Concentrations and
Relation to Birthweight, Head Circumference and Infant Length at Age 14
Days. Paediatr Perinat Epidemiol.

[r6] Dressler N, Chandra A, Aguirre Davila L, Spineli LM, Schippert C, von Versen-Hoynck F (2016). BMI and season are associated with vitamin D deficiency in women
with impaired fertility: a two-centre analysis. Arch Gynecol Obstet.

[r7] Fabris A, Pacheco A, Cruz M, Puente JM, Fatemi H, Garcia-Velasco JA (2014). Impact of circulating levels of total and bioavailable serum
vitamin D on pregnancy rate in egg donation recipients. Fertil Steril.

[r8] Farzadi L, Khayatzadeh Bidgoli H, Ghojazadeh M, Bahrami Z, Fattahi A, Latifi Z, Shahnazi V, Nouri M (2015). Correlation between follicular fluid 25-OH vitamin D and assisted
reproductive outcomes. Iran J Reprod Med.

[r9] Firouzabadi RD, Rahmani E, Rahsepar M, Firouzabadi MM (2014). Value of follicular fluid vitamin D in predicting the pregnancy
rate in an IVF program. Arch Gynecol Obstet.

[r10] Harel Z, Flanagan P, Forcier M, Harel D (2011). Low vitamin D status among obese adolescents: prevalence and
response to treatment. J Adolesc Health.

[r11] Holick MF, Binkley NC, Bischoff-Ferrari HA, Gordon CM, Hanley DA, Heaney RP, Murad MH, Weaver CM, Endocrine Society (2011). valuation, treatment, and prevention of vitamin D deficiency: an
Endocrine Society clinical practice guideline. J Clin Endocrinol Metab.

[r12] LeBlanc ES, Zakher B, Daeges M, Pappas M, Chou R (2015). Screening for vitamin D deficiency: a systematic review for the
U.S. Preventive Services Task Force. Ann Intern Med.

[r13] Luk J, Torrealday S, Neal Perry G, Pal L (2012). Relevance of vitamin D in reproduction. Hum Reprod.

[r14] Lv SS, Wang JY, Wang XQ, Wang Y, Xu Y (2016). Serum vitamin D status and in vitro fertilization outcomes: a
systematic review and meta-analysis. Arch Gynecol Obstet.

[r15] Ozkan S, Jindal S, Greenseid K, Shu J, Zeitlian G, Hickmon C, Pal L (2010). Replete vitamin D stores predict reproductive success following
in vitro fertilization. Fertil Steril.

[r16] Paffoni A, Ferrari S, Vigano P, Pagliardini L, Papaleo E, Candiani M, Tirelli A, Fedele L, Somigliana E (2014). Vitamin D deficiency and infertility: insights from in vitro
fertilization cycles. J Clin Endocrinol Metab.

[r17] Panda DK, Miao D, Tremblay ML, Sirois J, Farookhi R, Hendy GN, Goltzman D (2001). Targeted ablation of the 25-hydroxyvitamin D 1alpha -hydroxylase
enzyme: evidence for skeletal, reproductive, and immune
dysfunction. Proc Natl Acad Sci U S A.

[r18] Perez-Lopez FR, Pasupuleti V, Mezones-Holguin E, Benites-Zapata VA, Thota P, Deshpande A, Hernandez AV (2015). Effect of vitamin D supplementation during pregnancy on maternal
and neonatal outcomes: a systematic review and meta-analysis of randomized
controlled trials. Fertil Steril.

[r19] Polyzos NP, Anckaert E, Guzman L, Schiettecatte J, Van Landuyt L, Camus M, Smitz J, Tournaye H (2014). Vitamin D deficiency and pregnancy rates in women undergoing
single embryo, blastocyst stage, transfer (SET) for IVF/ICSI. Hum Reprod.

[r20] Reis JP, von Muhlen D, 3rd Miller ER, Michos ED, Appel LJ (2009). Vitamin D status and cardiometabolic risk factors in the United
States adolescent population. Pediatrics.

[r21] Rudick B, Ingles S, Chung K, Stanczyk F, Paulson R, Bendikson K (2012). Characterizing the influence of vitamin D levels on IVF
outcomes. Hum Reprod.

[r22] Tabesh M, Salehi-Abargouei A, Tabesh M, Esmaillzadeh A (2013). Maternal vitamin D status and risk of pre-eclampsia: a systematic
review and meta-analysis. J Clin Endocrinol Metab.

[r23] Terushkin V, Bender A, Psaty EL, Engelsen O, Wang SQ, Halpern AC (2010). Estimated equivalency of vitamin D production from natural sun
exposure versus oral vitamin D supplementation across seasons at two US
latitudes. J Am Acad Dermatol.

[r24] Theodoratou E, Tzoulaki I, Zgaga L, Ioannidis JP (2014). Vitamin D and multiple health outcomes: umbrella review of
systematic reviews and meta-analyses of observational studies and randomised
trials. BMJ.

[r25] Wortsman J, Matsuoka LY, Chen TC, Lu Z, Holick MF (2000). Decreased bioavailability of vitamin D in obesity. Am J Clin Nutr.

